# Early Changes in [^18^F]FDG Uptake as a Readout for PI3K/Akt/mTOR Targeted Drugs in HER-2-Positive Cancer Xenografts

**DOI:** 10.1155/2021/5594514

**Published:** 2021-05-25

**Authors:** Yanina Dockx, Christel Vangestel, Tim Van den Wyngaert, Manon Huizing, Sven De Bruycker, Patrick Pauwels, Steven Staelens, Sigrid Stroobants

**Affiliations:** ^1^Molecular Imaging Center Antwerp, University of Antwerp, Universiteitsplein 1, B-2610 Wilrijk, Belgium; ^2^Nuclear Medicine, Antwerp University Hospital, Wilrijkstraat 10, B-2650 Edegem, Belgium; ^3^Medical Oncology, Antwerp University Hospital, Wilrijkstraat 10, B-2650 Edegem, Belgium; ^4^Anatomical Pathology, Antwerp University Hospital, Wilrijkstraat 10, B-2650 Edegem, Belgium

## Abstract

We investigated the potential use of [^18^F]FDG PET as a response biomarker for PI3K pathway targeting therapies in two HER-2-overexpressing cancer models. *Methods*. CD-1 nude mice were inoculated with HER-2-overexpressing JIMT1 (trastuzumab-resistant) or SKOV3 (trastuzumab-sensitive) human cancer cells. Animals were treated with trastuzumab, everolimus (mTOR inhibitor), PIK90 (PI3K inhibitor), saline, or combination therapy. [^18^F]FDG scans were performed at baseline, two, and seven days after the start of the therapy. Tumors were delineated on CT images and relative tumor volumes (RTV) and maximum standardized uptake value (SUV_max_) were calculated. Levels of pS6 and pAkt on protein tumor lysates were determined with ELISA. *Results*. In the SKOV3 xenografts, all treatment schedules resulted in a gradual decrease in RTV and delta SUV_max_ (*Δ*SUV_max_). For all treatments combined, *Δ*SUV_max_ after 2 days was predictive for RTV after 7 days (*r* = 0.69, *p* = 0.030). In JIMT1 tumors, monotherapy with everolimus or PIK90 resulted in a decrease in RTV (−30% ± 10% and −20% ± 20%, respectively) and *Δ*SUV_max_ (−39% ± 36% and −42% ± 8%, respectively) after 7 days of treatment, but not earlier, while trastuzumab resulted in nonsignificant increases compared to control. Combination therapies resulted in RTV and *Δ*SUV_max_ decrease already at day 2, except for trastuzumab+everolimus, where an early flare was observed. For all treatments combined, *Δ*SUV_max_ after 2 days was predictive for RTV after 7 days (*r* = 0.48, *p* = 0.028), but the correlation could be improved when combination with everolimus (*r* = 0.59, *p* = 0.023) or trastuzumab (*r* = 0.69, *p* = 0.015) was excluded. *Conclusion*. Reduction in [^18^F]FDG after 2 days correlated with tumor volume changes after 7 days of treatment and confirms the use of [^18^F]FDG PET as an early response biomarker. Treatment response can however be underestimated in schedules containing trastuzumab or everolimus due to temporary increased [^18^F]FDG uptake secondary to negative feedback loop and crosstalk between different pathways.

## 1. Introduction

**Figure 1:**
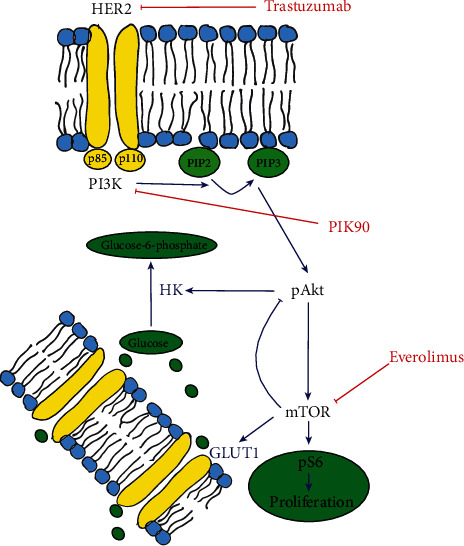
Schematic overview of the regulation of glucose metabolism by the PI3K/Akt/mTOR pathway. The PI3K pathway is initiated when a growth factor like HER-2 binds its receptor. PI3K then catalyzes the phosphorylation of phosphatidylinositol biphosphatase (PIP2) to phosphatidylinositol triphosphatase (PIP3). PIP3 recruits and activates Akt. Thereafter, phosphorylated Akt stimulates mTOR. In the cell, Akt stimulates HK; as a consequence, glucose and [^18^F]FDG are phosphorylated at a higher rate. mTOR has an effect on the glucose metabolism through upregulation of the expression of GLUT1. Trastuzumab is an anti-HER-2 humanized monoclonal antibody. PIK90 is a PI3K inhibitor and everolimus is an mTOR inhibitor.

Trastuzumab is a monoclonal antibody directed against human epidermal growth factor 2 (HER-2), which is overexpressed in 25% of breast cancers [1]. Despite the selection of HER-2-overexpressing tumors, only a minority of trastuzumab-treated patients respond to therapy. Relapse is often associated with drug resistance. This observed resistance has been linked to the aberrant activation of the phosphatidylinositol-3-kinase/Akt/mammalian target of rapamycin (PI3K/Akt/mTOR) pathway (Figure 1), in which Akt and mTOR are the major effector kinases. Inhibition of this signaling cascade has been a focus in the development of new therapeutics to overcome trastuzumab resistance, such as PI3K inhibitors (e.g., PIK90) and mTOR inhibitors (e.g., everolimus), which act on different levels in the pathway. An important observation after mTOR blockade is the increase in pAkt due to the activation of alternative pathways and/or downregulation of negative feedback loops resulting in suboptimal inhibition of the pathway [2]. Although targeted therapies are overall better tolerated than standard chemotherapy, they still cause side effects and are quite expensive. Therefore, the ability to select nonresponders early would enable to modify the therapeutic strategies early in the treatment course.

To date, clinical tumor biomarkers that accurately predict or monitor tumor response to PI3K/Akt/mTOR targeted drugs are not available. The ability to monitor response earlier during therapy would allow unresponsive patients to receive alternative therapy and reduce the cost to the healthcare system. The criteria to evaluate response are based upon morphological imaging and are available at the earliest two months following effective therapy. Moreover, tissue biopsies do not adequately represent the heterogeneity of tumors and cannot be sampled longitudinally. Imaging modalities such as positron emission tomography (PET) provide an attractive option, since such techniques generate information about the whole tumor burden. PET assesses the glucose metabolism of cancer with the glucose analogue ^18^F-fluorodeoxyglucose ([^18^F]FDG).

Several clinical studies in breast cancer confirmed the use of [^18^F]FDG PET as an early response biomarker [3, 4], but results in HER-2-overexpressing patients are less clear. The Neo-ALTTO study showed that patients achieving a pathological complete response (pCR) after 12 weeks of neoadjuvant HER-2 targeted treatment (trastuzumab, lapatinib, or combination) have more pronounced reductions in [^18^F]FDG uptake after 2 and 6 weeks of treatment compared to those patients with residual disease [3, 5]. In contrast, both Koolen et al. [6] and Cheng et al. [7] reported that early [^18^F]FDG PET predicts pCR only in HER-2-negative patients, but not in HER-2-positive patients treated with neoadjuvant chemotherapy containing trastuzumab. Finally, it still needs to be proven that PET response adapted therapy will ultimately improve survival. In the AVATAXHER study, PET response after 2 cycles of docetaxel-trastuzumab was associated with higher pCR rates, but the addition of bevacizumab in PET nonresponders did not result in a significant improvement in disease-free survival [8]. Also, the role of [^18^F]FDG to predict response after PI3K/Akt/mTOR targeted treatment is still a matter of debate. The PI3K inhibitor alpelisib is currently available in the clinic in combination with fulvestrant for postmenopausal women with advanced or metastatic hormone receptor-positive, HER-2-negative, PIK3CA-mutated breast cancer [9]. [^18^F]FDG PET demonstrated dose-dependent changes after PI3K inhibition as a pharmacodynamic biomarker [10, 11]. Decreases in [^18^F]FDG uptake after anti-PI3K treatment confirmed target modulation; however, no correlation was found between molecular alterations and clinical activity [12]. A phase I study in breast, endometrial, and ovarian cancer evaluated [^18^F]FDG as a biomarker after therapy with an mTOR inhibitor (temsirolimus) and pegylated liposomal doxorubicin and [^18^F]FDG PET was able to predict early response [13]. Another clinical study, where breast cancer patients were treated with everolimus and exemestane, early [^18^F]FDG PET 14 days after the start of therapy could identify patients at high risk of nonresponse [14]. These results contradict a preclinical investigation, in which everolimus inhibited tumor growth in relative insensitive (human colon and cervical) and sensitive (human melanoma and lung cancer) mTOR cancer models; tumor [^18^F]FDG uptake after 2 and 7 days of treatment was only reduced in sensitive cell lines. It was concluded that [^18^F]FDG was not predictive of a proliferative response to mTOR inhibition [15].

Possible explanations for the discrepancies in the results are twofold. Firstly, an inflammatory response can be induced by trastuzumab treatment, masking the response prediction by [^18^F]FDG PET [6, 7]. Secondly, mTOR inhibitors have, beside antiproliferative effects, also antiangiogenic and proapoptotic properties, which do not always lead to [^18^F]FDG reduction and could provide false-negative results [15].

As a consequence, it is unknown if [^18^F]FDG PET is a good readout of cell kill. Therefore, we investigated if the decreased viability of HER-2-overexpressing tumors exposed to targeted PI3K/Akt/mTOR drugs would diminish their glucose utilization and [^18^F]FDG PET uptake. We assessed [^18^F]FDG PET uptake and tumor volume in trastuzumab-sensitive and trastuzumab-resistant HER-2-overexpressing tumor xenografts after treatment for 2 and 7 days with PI3K/Akt/mTOR targeted drugs. We verified if [^18^F]FDG PET was able to early and correctly detect the effect of treatment.

## 2. Material and Methods

### 2.1. Products, Cell Lines, and Animal Models

Everolimus and trastuzumab were generous gifts from Novartis (Basel Switzerland) and Roche (Basel Switzerland), respectively. PIK90 was purchased from Selleckchem (Houston, TX, USA).

The human breast adenocarcinoma cell line JIMT1 (HER-2 overexpressing, resistant to trastuzumab, sensitive to everolimus and PIK90; Deutsche Sammlung von Mikroorganismen und Zellkulturen, Germany) and the human ovarian cancer cell line SKOV3 (HER-2 overexpressing, sensitive to all tested drugs; American Type Culture Collection (number HTB-77), Manassas, VA, USA) were cultured according to supplier's instructions. Short tandem repeat (STR) profiling and HER-2 fluorescence *in situ* hybridization (FISH) was performed.

Female CD1 athymic nude mice (*n* = 102; Charles Rivers, Calco, Italy) were purchased at the age of 6 weeks. Animals were group-housed (6 per cage) in individually ventilated cages under 12 : 12 dark/light cycle, controlled temperature (20-23°C) and humidity (50-60%) with *ad libitum* access to standard laboratory chow and water.

Suspensions of 5 × 10^6^ viable tumor cells in 0.1 ml PBS were inoculated orthotopically into the lower mammary fat pad (JIMT1) or subcutaneously into the lower limb (SKOV3). Tumor growth was evaluated three times a week with digital caliper measurements from the moment tumors became palpable. Tumor volume was calculated with the formula *V* = 0.5 × (length × width^2^). Treatment was initiated 4 to 6 weeks after inoculation, when the tumors reached a size of approximately 100 mm^3^. All mice with a tumor volume less than 75 mm^3^ at the start of the experiment were excluded from the analyses. The experimental protocol was approved by the Antwerp University Ethical Committee for Animal Experiments, and all applicable institutional and European guidelines for the care and use of animals were followed.

### 2.2. Study Design

**Figure 2:**
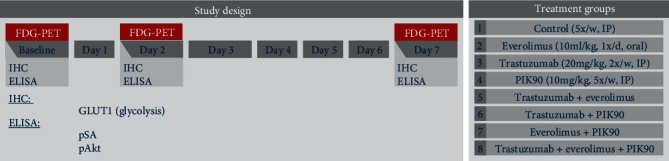
Schedule of imaging experiments, *ex vivo* validations on excised tumor tissue and therapy scheme. Thirty days after inoculation of JIMT1 and SKOV3 cells, mice underwent an [^18^F]FDG PET scan. Longitudinal [^18^F]FDG PET imaging was performed at baseline (day 0) and after start of therapy on day 2 (d2) and on day 7 (d7). After the baseline scans, treatment was initiated with control, trastuzumab, everolimus, or PIK90. The combination groups received trastuzumab+everolimus or trastuzumab+PIK90 or everolimus+PIK90 or trastuzumab+everolimus+PIK90.

Tumor-bearing mice were randomized into 8 different treatment groups. A schematic representation of the setup of the imaging study can be found in Figure 2. In the trastuzumab group, therapy was administered twice a week (Monday and Thursday) in a concentration of 20 mg/kg intraperitoneal (IP). Everolimus was administered through oral gavage daily at a concentration of 10 ml/kg. In the third monotherapy group, PIK90 was administered five times a week (Monday to Friday) IP in a concentration of 10 mg/kg. The combination groups received trastuzumab+everolimus or trastuzumab+PIK90 or everolimus+PIK90 or trastuzumab+everolimus+PIK90 (same doses as listed above). Control animals received an IP injection with vehicle (saline). Relative tumor volume (RTV) was calculated as *V*_*x*_/*V*_1_, with *V*_*x*_, volume at time point *t*, and *V*_1_, the volume at baseline, reported as percentages.

### 2.3. [^18^F]FDG PET/CT Imaging

Longitudinal [^18^F]FDG PET imaging was performed at baseline (day 0) and after the start of therapy on day 2 (d2), and on day 7 (d7). In total, 9 tumor-bearing mice were included in the control group and 6 tumor-bearing mice in each treatment group for each cell line. Three control animals were sacrificed at d0, and three animals per group were sacrificed at d2 and d7 by cervical dislocation to obtain tumor tissue for correlative *ex vivo* analyses (Figure 2).

[^18^F]FDG was synthesized at the Department of Nuclear Medicine of the Antwerp University Hospital, using a GE FASTlab synthesizer (GE Healthcare, Diegem, Belgium). For all [^18^F]FDG experiments, animals were fasted overnight. Previous studies have reported the effects of fasting time, anesthetic agents, and blood glucose levels on the uptake of [^18^F]FDG in mice [16]. To minimize variability, all mice were handled under the same fasting state, anesthetic agent, incubation time, and body temperature.

For JIMT1 xenografts, bladder flushing was performed to prevent the accumulation of radiotracer in the bladder during the scan. Since the orthotopic tumor is located in close vicinity to the bladder and [^18^F]FDG clears renally, the bladder activity can mask tumor uptake and therefore needs to be void of radioactive urine. A double-lumen urethral catheter optimized for molecular imaging was inserted to be able to flush the bladder continuously during PET acquisition, as previously described [17].

Approximately 18.5 MBq [^18^F]FDG in a final volume of 200 *μ*l was injected via the tail vein of anesthetized mice. Prior to tracer administration, whole blood glucose levels were measured from a blood drop obtained from the contralateral tail vein using an OneTouch Ultra 2 glucose meter (LifeScan, Tilburg, The Netherlands). Glucose measurements were performed in duplicate, with an average value of 94 ± 19 mg/dl. CT acquisition, which was performed for attenuation and scatter correction purposes, was followed by static PET acquisition on an Inveon small-animal PET-CT scanner (Siemens Preclinical Solution, Knoxville, TN, USA).

PET images were reconstructed using 4 iterations × 16 subsets of a 3-dimensional ordered subset expectation maximization (OSEM3D) algorithm following Fourier rebinning. Normalization as well as correction for dead time, scatter, and attenuation was applied. Using PMOD v3.3 software (PMOD Technologies, Zurich, Switzerland), regions of interest (ROI) were drawn on the CT images manually for qualitative assessment covering the whole tumor. Maximum standardized uptake values (SUV_max_) were calculated. SUV_max_ was used to correct for tumor heterogeneity due to central necrosis. Delta SUV_max_ (*Δ*SUV_max_) was calculated as SUV_max,*t*_/SUV_max,1_, with SUV_max,*t*_ SUV_max_ at time point *t* and SUV_max,1_ SUV_max_ at baseline, visualized as percentages.

### 2.4. Ex Vivo Validation

At the end of the study, animals were sacrificed and tumor tissue was resected. One part was formalin-fixed and paraffin-embedded for histological examination by immunohistochemistry (IHC) for glucose transporter 1 (GLUT1). Tissue sections of 3 *μ*m thick were mounted on SuperFrost microscope slides (Menzel-Glaser, Braunschweig, Germany). IHC was performed according to the manufacturers' instructions. The following antibody was used: #ab652 (Abcam, Cambridge, UK; 1 : 250; 45 minutes incubation at room temperature). After washing, sections were incubated with anti-rabbit horseradish peroxidase- (HRP-) labeled secondary antibody (Dako, Santa Clara, CA, USA; ready-to-use). Negative controls were incubated with antibody diluent instead of the primary antibody.

IHC slices were examined by means of a CX31 microscope (Olympus, Aartselaar, Belgium). Only distinct membrane staining was scored as positive. Intensity was graded as absent, score 0; mild, score 1; moderate, score 2; or intense, score 3. The percentage of positive tumor cells was scored as follows: 0%, score 0; 1-20%, score 1; 21-40%, score 2; 41-60%, score 3; 61-80%, score 4; or 81-100%, score 5. A final histological score (*H*-score) was calculated after counting 10 high power fields as follows: *H*‐score = [(*a*1 × *i*1) + (*a*2 × *i*2)]/2, with *a* and *i* being the amount and the intensity of positively stained tumor cells, respectively, and 1 and 2 refer to 2 independent observers [18].

The other part of the tumor was snap-frozen in liquid nitrogen and stored at -80°C. Tumor tissues were homogenized using a GentleMACS tissue dissociator (Miltenyi Biotec, Bergisch Gladbach, Germany). Radioimmunoprecipitation assay (RIPA-buffer together with 1% antifoam Y-30 (Thermo Fisher Scientific, Waltham, MA, USA)) was used to obtain protein lysates from tumor tissue. Total protein content of the tumor tissue samples was measured using the bicinchoninic acid (BCA) Protein Assay Kit (Thermo Fisher Scientific), according to the provided protocol. The pAkt and pS6 ELISA were performed using the PathScan Phospho-Akt1 Ser473 Sandwich ELISA kit (Cell Signaling, Leiden, The Netherlands) and the pS6 PathScan Phospho-S6 Ser 235/236 Sandwich ELISA kit (Cell Signaling), respectively, according to the manufacturer's guidelines. All samples were run in duplicate in appropriate dilutions. All ELISA data were normalized for protein amount.

### 2.5. Statistical Analysis

Statistical analyses were performed using SPSS software v20 (IBM Corp., Chicago, IL, USA). Repeated measures ANOVA was conducted for analyzing [^18^F]FDG uptake and RTV between the different treatment groups during the imaging study. Differences between unmatched groups were analyzed using Kruskal-Wallis and Mann–Whitney *U* tests. Spearman's correlation analyses were used to investigate correlations between measurements. *p* ≤ 0.05 was considered as statistically significant. Results from the *in vivo* experiments are expressed as mean ± standard error mean (SEM). Results from the *ex vivo* experiments are expressed as mean ± standard deviation (SD).

## 3. Results

### 3.1. Effect of Monotherapies on Tumor Growth and [^18^F]FDG Uptake

For both xenografts, no significant differences in initial tumor volume could be observed between mice of the different treatment groups before treatment was initiated (data not shown). In addition, baseline images showed similar [^18^F]FDG SUV_max_ in tumors of the different mice in JIMT1 xenografts (1.81 ± 0.59) and SKOV3 xenografts (1.45 ± 0.36).

**Figure 3:**
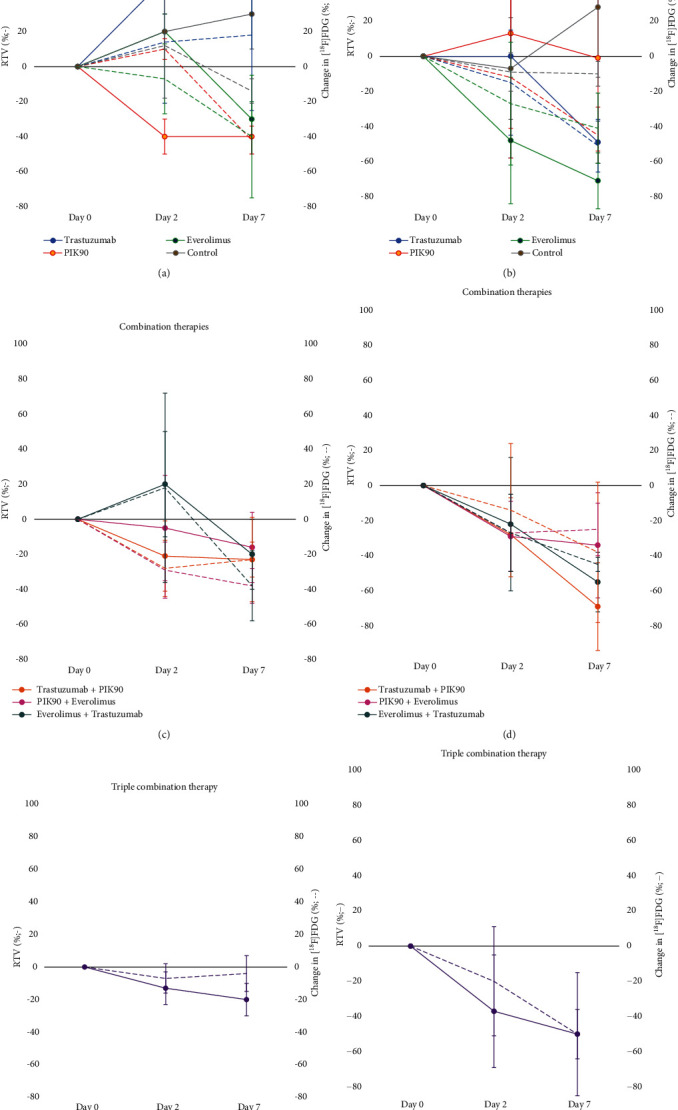
*In vivo* longitudinal imaging study of JIMT1 and SKOV3 xenografts. Graphic representation of tumor growth curves and relative [^18^F]FDG SUV_max_ at day 2 and day 7 of treatment for (a, c, e) JIMT1 and (b, d, f) SKOV3 xenografts.

In trastuzumab-resistant JIMT1 tumors, 7 days of treatment with everolimus or PIK90 monotherapy resulted in a decrease in RTV of −30 ± 10% and −20 ± 20%, respectively (Figure 3(a)). In concordance, similar decreases in *Δ*SUV_max_ were observed of, respectively, −39 ± 36% and −42 ± 8% (*p* = 0.030) (Figure 3(a)). No major changes in RTV or *Δ*SUV_max_ were observed at day 2, except for RTV after PIK90 (−40 ± 10%). As expected, monotherapy with trastuzumab did not result in significant volume or SUV changes in this resistant cell line, but nonsignificant increases compared to control could be observed.

n trastuzumab-sensitive SKOV3 xenografts, trastuzumab and everolimus resulted in a pronounced reduction in RTV (−49 ± 12% (*p* = 0.050) and −71 ± 16% (*p* = 0.050), respectively) and *Δ*SUV_max_ (−51 ± 15% (*p* = 0.035) and −41 ± 20% (*p* = 0.010), respectively) after 7 days of treatment. Surprisingly, no change in RTV was observed after PIK90, while *Δ*SUV_max_ was decreased with −45 ± 16% (Figure 3(b)). No major reductions in RTV were observed after 2 days of treatment except for everolimus, while *Δ*SUV_max_ was already slightly reduced for all treatments. Indeed, the major reduction in RTV (−48 ± 36%) compared to *Δ*SUV_max_ (−27 ± 35%) is remarkable.

### 3.2. Effect of Combination Therapies on Tumor Growth and [^18^F]FDG Uptake

In JIMT1 xenografts, significant reductions in RTV and *Δ*SUV_max_ were observed after 7 days of treatment with all dual treatment combinations (Figure 3(c)). In everolimus-containing regimens, reductions in *Δ*SUV_max_ were more pronounced compared to RTV. Combining trastuzumab with PIK90 or everolimus resulted in smaller decreases in RTV and *Δ*SUV_max_ compared to monotherapy with PIK90 or everolimus. Temporal changes of RTV and *Δ*SUV_max_ were comparable with a small flare at day 2 for trastuzumab+everolimus and early reduction after trastuzumab+PIK90. In contrast, no early RTV change was observed after PIK90+everolimus, whereas *Δ*SUV_max_ was already substantially reduced (38% ± 10%). Remarkably, whereas triple combination therapy induced a similar decrease in RTV at day 7 compared with doublets, *Δ*SUV_max_ remained unchanged (−4 ± 10%, Figure 3(e)). In SKOV3 xenografts, all treatment schedules resulted in a similar and gradual decrease in RTV and *Δ*SUV_max_ (Figure 3(d)). Triple therapy resulted in the highest *Δ*SUV_max_ decrease (−50 ± 14%, *p* = 0.007, Figure 3(f)).

**Figure 4:**
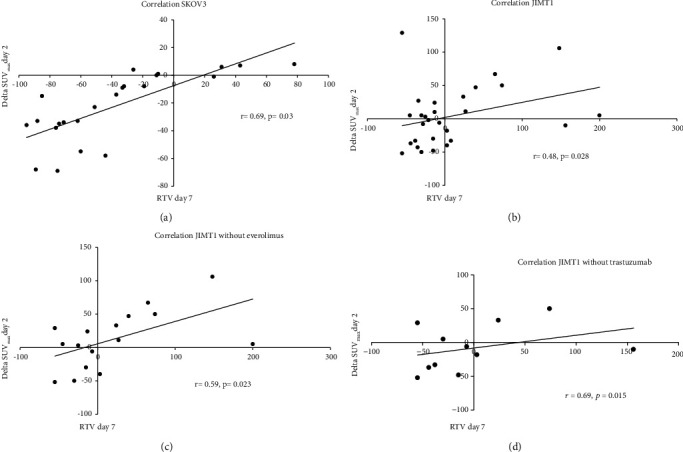
Correlation between volume and metabolic activity. Correlation of RTV at day 7 with delta SUV_max_ at day 2 for (a) SKOV3, (b) JIMT1, (c) without everolimus, (d) without trastuzumab.

To evaluate if changes in the [^18^F]FDG response predict volume changes, *Δ*SUV_max_ after 2 days was correlated with RTV after 7 days. For all treatments combined, a moderate correlation was found in SKOV3 xenografts (*r* = 0.69, *p* = 0.030) and a low correlation was found in JIMT1 xenografts (*r* = 0.48, *p* = 0.028) (Figures 4(a) and 4(b)). In JIMT1, the correlation could be improved when combinations with everolimus treatment (*r* = 0.59, *p* = 0.023) or trastuzumab (*r* = 0.69, *p* = 0.015) were excluded (Figures 4(c) and 4(d)).

**Figure 5:**
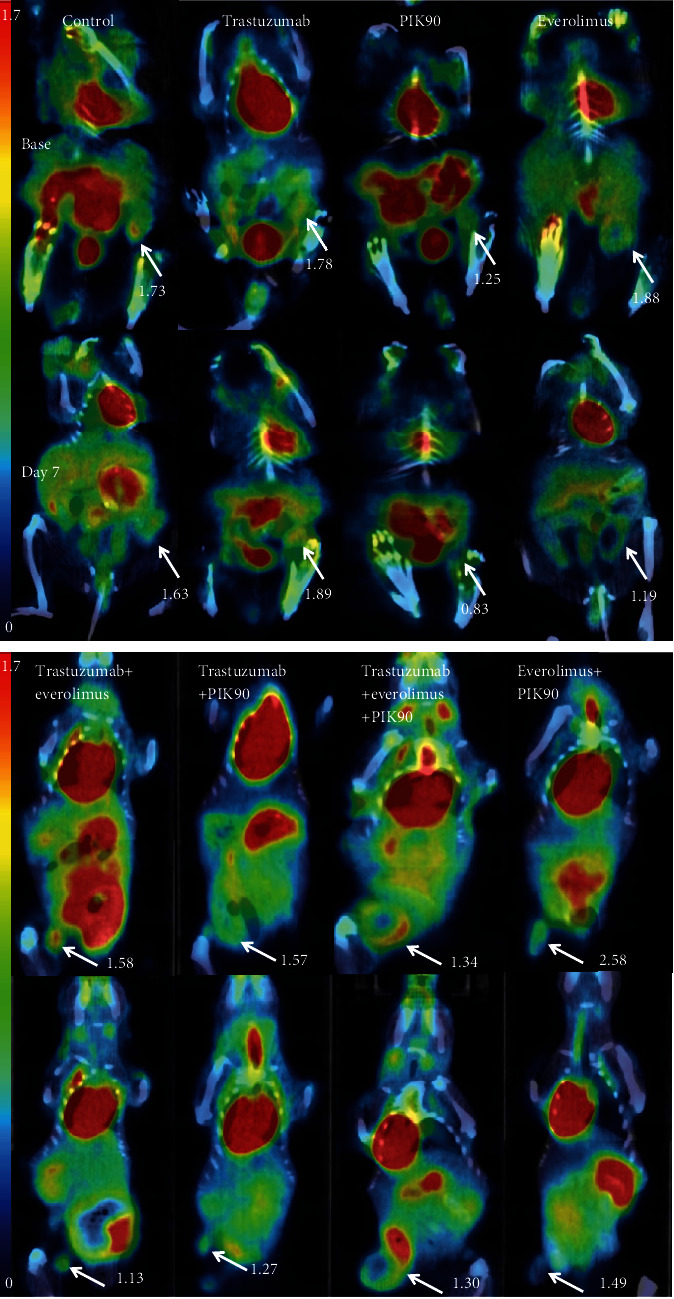
Representative images of JIMT1 xenografts. Representative [^18^F]FDG PET/CT SUV-corrected images of mice exposed to monotherapy and combination therapy at baseline and after day 7. White arrows indicate tumors with the SUV_max_ of that tumor. Mice treated with monotherapy are imaged in prone position and mice treated with combination therapy in supine position.

**Figure 6:**
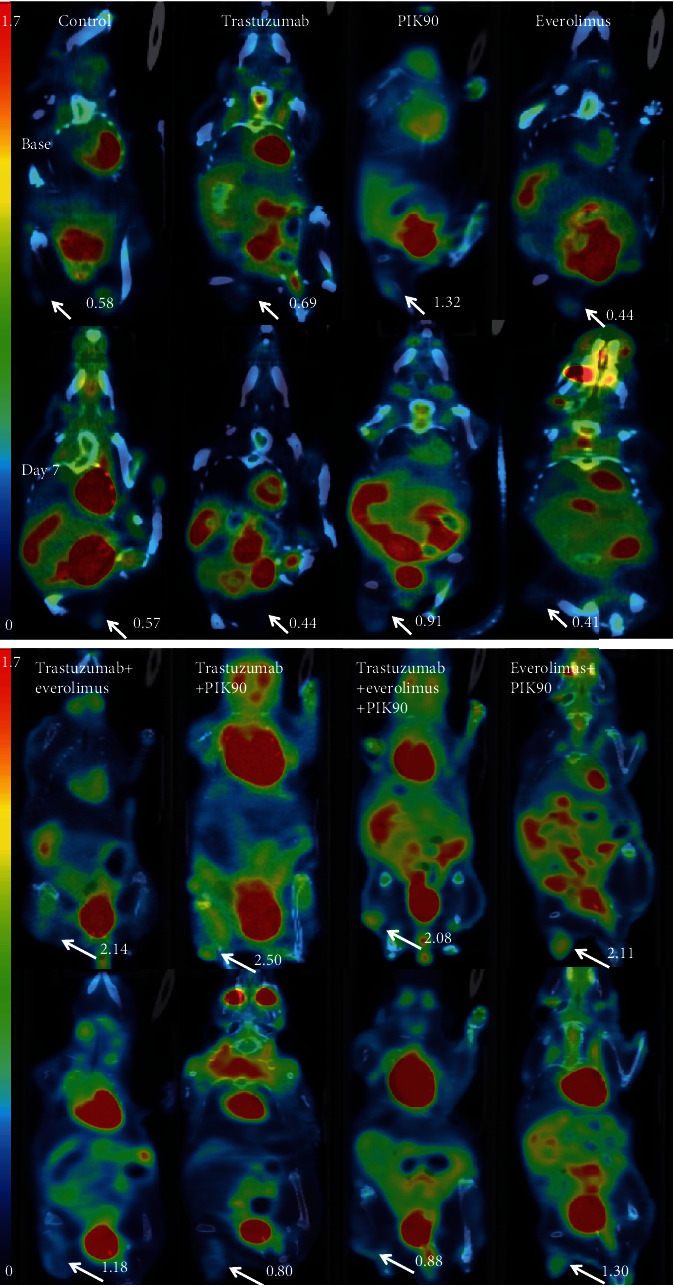
Representative images of SKOV3 xenografts. Representative [^18^F]FDG PET/CT SUV-corrected images of mice exposed to mono- and combination therapy at baseline and after day 7. White arrows indicate tumors with SUV_max_ of that tumor. All mice were imaged in supine position.

Representative [^18^F]FDG *μ*PET/CT images of JIMT1 and SKOV3 xenografts treated with vehicle, monotherapy, or combination therapies are shown in Figures 5 and 6, respectively.

### 3.3. [^18^F]FDG Uptake and PI3K/Akt/mTOR Pathway Inhibition

**Figure 7:**
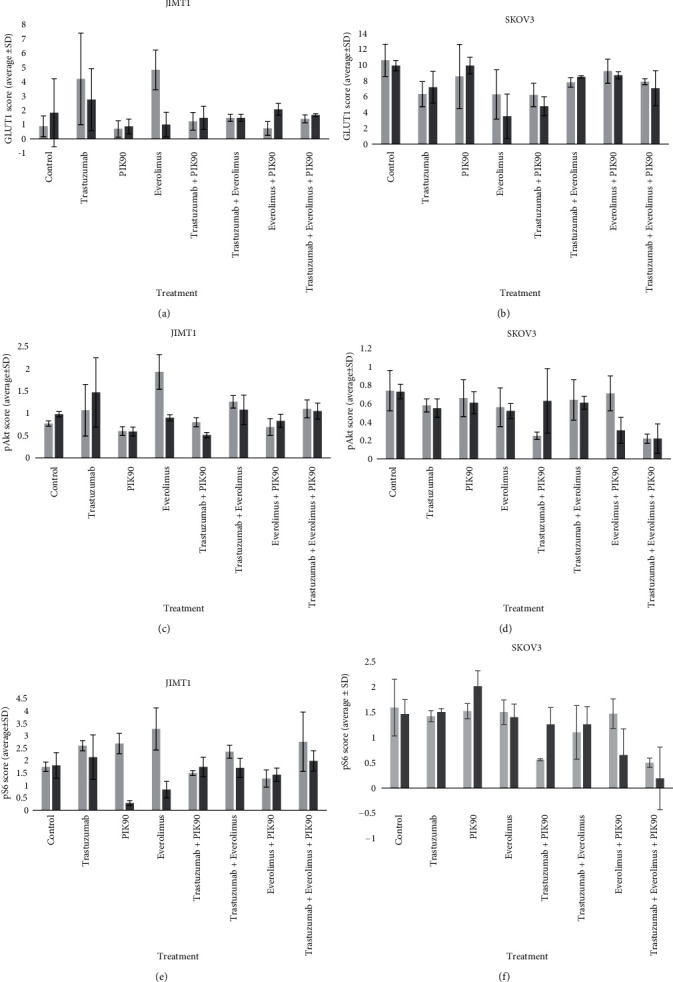
*Ex vivo* longitudinal imaging study of JIMT1 and SKOV3 xenografts. Graphic representation of GLUT1, pAkt, and pS6 at day 2 (light grey) and day 7 (dark grey) of treatment for (a, c, e) JIMT1 and (b, d, f) SKOV3 xenografts.

In JIMT1, monotherapy with everolimus and PIK90 inhibited the pathway downstream, exemplified by a significant decrease in pS6 of, respectively, 0.83 ± 0.33 (*p* = 0.036) and 0.29 ± 0.10 (*p* = 0.034), at day 7, but not earlier (Figure 7(e)). In contrast, after trastuzumab therapy, an increase in pS6 was seen after 2 days (2.60 ± 0.21, *p* = 0.039) compared to control and combining trastuzumab with one or both other drugs abolished the pS6 reduction. Early increase in pAkt was observed after everolimus monotherapy (1.93 ± 0.39, *p* = 0.022, Figure 7(c)) or combined with trastuzumab (1.26 ± 0.14) in comparison to the control sample (0.77 ± 0.06), whereas PIK90 alone (0.59 ± 0.10, *p* = 0.006) or combined with trastuzumab (0.51 ± 0.06) reduced pAkt at day 2 and day 7.

As [^18^F]FDG uptake is closely related to GLUT expression on the cell membrane, but the latter is also regulated by the PI3K pathway, treatment-induced changes of GLUT1 were evaluated and correlated with pAkt and pS6. Surprisingly, we only observed a significant early increase in GLUT1 after everolimus (4.83 ± 1.39, *p* = 0.002, Figure 7(a)) and a sustained although not significant increase after trastuzumab. The latter could explain the increased uptake or “flare” after [^18^F]FDG uptake during trastuzumab monotherapy, but overall, no significant correlations between GLUT1 expression and [^18^F]FDG uptake nor with GLUT1, pAkt, or pS6 were found. Of note is the weak baseline expression of GLUT1, which suggests that it is likely that other GLUTs play a role in [^18^F]FDG uptake. To evaluate if [^18^F]FDG uptake was readout of pathway inhibition, the potential correlation of SUV_max_ with pAkt and pS6 after 2 and 7 days of treatment was evaluated. pS6 only was only significantly correlated with [^18^F]FDG uptake when trastuzumab treatment was excluded (*r* = 0.75, *p* = 0.040).

In SKOV3 xenografts, monotherapy was not able to reduce pAkt and pS6 levels; pS6 was even slightly increased after 7 days of PIK90 therapy (Figures 7(d) and 7(f)). Pathway inhibition was seen after combination therapies and was most pronounced in PIK90 containing regimens, but only triple combination treatment was able to reduce pAkt and pS6 levels significantly both after 2 days (0.22 ± 0.05 (*p* = 0.009) and 0.50 ± 0.09 (*p* = 0.016)) and 7 days of treatment (0.22 ± 0.16 (*p* = 0.050) and 0.19 ± 0.62 (*p* = 0.020)). Reduction in GLUT1 expression was observed after all treatments but only reached significance 7 days after monotherapy with everolimus (3.53 ± 2.81, *p* = 0.020) and 2 days after all combination treatments with trastuzumab (Figure 7(b)). GLUT1 expression correlated with pAkt (*r* = 0.22, *p* = 0.009) and was closely related to [^18^F]FDG uptake; consequently, SUV_max_ correlated with both pAkt (*r* = 0.57, *p* = 0.020) and pS6 (*r* = 0.7, *p* = 0.030).

## 4. Discussion

New treatment options are needed for patients with HER-2-overexpressing tumors with de novo or acquired resistance to trastuzumab. Since resistance has been linked to the aberrant activation of the PI3K/Akt/mTOR pathway, combining trastuzumab with inhibitors of the major effector kinases Akt and mTOR is an attractive treatment strategy. Although the first clinical results are encouraging, biomarkers to identify patients who could derive the most benefit are urgently needed. Serial tumor biopsy samples for genomic or molecular analysis are invasive and rarely feasible in clinics. In contrast, noninvasive molecular imaging like PET is aimed at visualizing and quantifying cellular processes *in vivo*. Encouraged by previous observations [15, 19], PET may function as an early response biomarker for PI3K pathway targeting therapeutics. Imaging biomarkers ideally incorporate a dynamic system of measurements of response or failure and predict treatment outcomes.

In the current study, we evaluated the use of [^18^F]FDG PET as an early response biomarker in HER-2-overexpressing tumors treated with PI3K/Akt/mTOR-targeted therapy. The use of [^18^F]FDG PET for early response assessment is based on the assumption that metabolic changes precede tumor cell kill and volume reductions. We confirmed that changes in [^18^F]FDG uptake after 2 days correlate with tumor volume reductions after 7 days of treatment in both tumor models. Correlations were modest especially in the de novo trastuzumab-resistant xenograft JIMT1, treated with trastuzumab- or everolimus-containing schedules. Since the glucose metabolism and thus [^18^F]FDG uptake are partially regulated by key enzymes of the PI3K/Akt/mTOR pathway [20], treatment-induced temporary activation of pAkt and/or pS6 can have a direct pharmacodynamic effect on [^18^F]FDG uptake. In this study, we could indeed demonstrate that both trastuzumab and everolimus induced an early increase in pAkt and pS6 with corresponding increase in [^18^F]FDG uptake at day 2 in the JIMT1 xenograft. Due to this metabolic flare, no significant reduction in *Δ*SUV_max_ was observed after everolimus monotherapy and combinations with everolimus and trastuzumab, although these schedules induced a significant RTV reduction at day 7.

The potential of [^18^F]FDG PET as a predictive imaging biomarker for response to trastuzumab in HER-2-overexpressing breast cancer has been debated in previous studies. In the trastuzumab-sensitive SKOV3 xenograft, changes in *Δ*SUV_max_ did not precede volume reduction after trastuzumab monotherapy and this is in line with results of previous studies [21, 22]. The fact that trastuzumab acts more cytostatic than cytotoxic probably explains this slow metabolic response. As [^18^F]FDG uptake is considered a measure of cell viability, more specific PET tracers were investigated. In the trastuzumab-sensitive BT474 xenograft, Shah et al. [22] showed promising results with [^18^F]FLT-PET, a thymidine analogue to measure proliferation and optical imaging with NIR700-Annexin-V to evaluate apoptosis. While 1 week of trastuzumab had no effect on tumor volume or [^18^F]FDG uptake, a significant reduction in [^18^F]FLT and increase in NIR700-Annexin-V were observed.

**Figure 8:**
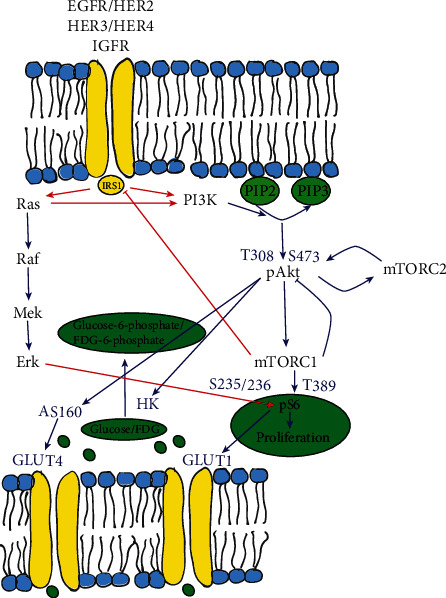
Schematic overview of the link between the PI3K/Akt/mTOR pathway and the Ras/MEK/ERK pathway. Growth factors bind receptor tyrosine kinases, which activate both the Ras/MEK/ERK and the PI3K/Akt/mTOR pathway, by regulating a cascade of phosphorylations. PI3K recruits and activates Akt on T308. Thereafter, phosphorylated Akt stimulates mTORC1 and mTORC2. Together with the phosphorylation of T308 by PI3K, S473 phosphorylation by mTORC2 is necessary for full Akt activation. After full activation of Akt, mTORC1 causes phosphorylation on T389 for pS6. mTORC1 initiates two negative feedback loops, firstly to Akt and secondly to IRS. Upon binding to receptor tyrosine kinases, the Ras/MEK/ERK pathway is also activated, eventually leading to activation of pS6 on S235/236. As known, activation of pS6 has an effect on cellular proliferation and glucose metabolism through upregulation of the expression of GLUT1.

Another puzzling finding was that trastuzumab appeared to stimulate tumor growth and [^18^F]FDG uptake in the resistant JIMT1 xenograft compared to controls, although differences were not significant. The HER-2-overexpressing JIMT1 cell line is intrinsically resistant to a variety of HER-2-targeting drugs, and the mechanisms of resistance have been unraveled over the last years. Besides an activating PIK3CA mutation that constitutively activates the pathway downstream, JIMT1 also displays an autocrine hyperactivation of the insulin receptor signaling with upregulated expression of the insulin-like growth factor I receptor (IGF-1R) and increased secretion of growth factors [23]. After ligand binding, IGF-1R activates a complex intracellular signaling network through insulin receptor substrate 1 (IRS1) proteins. The PI3K/Akt/mTOR and Ras/mitogen-activated extracellular signal-regulated kinase/extracellular signal-regulated kinase (Ras/MEK/ERK) pathways are the major downstream pathways of IGF-1R [24] (Figure 8). This phenomenon was nicely exemplified in a previous study in which pErk remained high after trastuzumab treatment in JIMT1 cells [25]. Although activation of IGF-1R is trastuzumab independent, exposure to trastuzumab can further stimulate IRS1 by heterodimerisation of HER-2 or HER-3 with IGF-1R [26]. When the trastuzumab-sensitive cell line SKBR3 was transfected to overexpress IGF-1R, trastuzumab exposure was found to stimulate proliferation to 125% [27]. The volume increase in JIMT1 after trastuzumab monotherapy observed in our study is thus in line with their findings. That this phenomenon can also result in increased [^18^F]FDG uptake was not reported before. Akt is not only a central hub in both the IGF-1R and HER-2 signaling cascade but also regulates the trafficking of GLUT1 and glucose transporter 4 (GLUT4) from cytoplasmatic vesicles to the cell membrane [28]. In our study, we found a sustained although not significant increase in pAkt and GLUT1 expression in JIMT1 after trastuzumab. Unfortunately, we did not measure GLUT4 expression as it is likely that changes in GLUT4 contribute significantly to the [^18^F]FDG uptake since GLUT1 expression was 5-fold lower at baseline in JIMT1 compared to the SKOV3 xenografts, while [^18^F]FDG uptake was higher. Moreover, GLUT1 and GLUT4 trafficking is regulated by different isoforms of Akt depending on the phosphorylation site S473 or T308. While GLUT1 expression requires phosphorylation on both T308 and S473, the latter regulated by mTORC2, phosphorylation of Akt on T308 is sufficient to activate AS160 for GLUT4 translocation and is IRS1-dependent [29]. Since IGF-1R is hyperactivated in JIMT1, it supports the role of GLUT4.

Similar to trastuzumab treatment, an early increase in [^18^F]FDG was also observed after everolimus treatment in JIMT1. Again, this observation can be explained by the presence of a negative feedback loop and the Ras/MEK/ERK pathway (Figure 8). Everolimus treatment leads to an upregulation of Akt through phosphorylation of S473 after mTORC2 assembly. Additionally, everolimus inhibits pS6 on T389, which activates the Ras/MEK/ERK pathway, eventually causing an increase in pS6 on S235/236 [30, 31]. These loops counteract the effect of everolimus and may explain why everolimus is cytostatic but not cytotoxic [32]. Indeed, in our xenograft models, everolimus caused a temporary increase of pAkt, pS6 (S235/236), and GLUT1 after 2 days. However, prolonged treatment with everolimus can also inhibit mTORC2 because of reduced availability of essential binding proteins due to progressive sequestration of the mTORC1 complex [33]. Inhibition of mTORC2 leads to inhibition of pS6 and eventually inhibition of GLUT1. As a consequence, [^18^F]FDG PET was only able to detect the response after 7 days of treatment. The same was true for the combination therapies. Whenever everolimus was included in the treatment regimen, the response was detected only at the latest time point, possibly because of the presence of these feedback loops.

The insights in the effect of the PI3K/Akt/mTOR pathway on glycolysis acquired in this study suggests the potential importance of feedback loops on the [^18^F]FDG signal (Figure 8). These effects are not only related to the drug itself but also influenced by acquired mutations that interfere with the crosstalk between important signaling cascades. We showed that early metabolic flare can result in an underestimation of the true response which emphasizes the importance of appropriate timing for PET response assessment.

## 5. Conclusion

[^18^F]FDG uptake after 2 days is correlated with tumor volume after 7 days of treatment and confirms the use of [^18^F]FDG PET as an early response biomarker for PI3K pathway targeting therapies. Treatment responses can, however, be underestimated due to an early metabolic flare, secondary to the crosstalk between the PI3K/Akt/mTOR pathway and glucose metabolism. In the JIMT1 model, both trastuzumab and everolimus induced a temporary stimulation of the PI3K/Akt/mTOR pathway with a corresponding increase in [^18^F]FDG uptake and therefore an incorrect or underestimation of the true response. These effects become less important at later time points when cell death comes to the fore. Therefore, future studies with new targeted therapies should refine imaging protocols with [^18^F]FDG, thereby critically considering the existence of feedback loops and timing.

## Data Availability

Data is available on request through the authors; correspondence: Sigrid Stroobants; e-mail: sigrid.stroobants@uza.be; Department of Nuclear Medicine, Antwerp University Hospital, Wilrijkstraat 10, B-2650 Edegem, Belgium; Tel: +32 3 821 35 68; Fax: +32 3 825 33 08.
